# PI3K inhibitor treatment ameliorates the glucocorticoid insensitivity of PBMCs in severe asthma

**DOI:** 10.1186/s40169-020-0262-5

**Published:** 2020-02-28

**Authors:** Jing Bi, Zhihui Min, Honglei Yuan, Zhilong Jiang, Ruolin Mao, Tao Zhu, Chunfang Liu, Yuzhen Zeng, Juan Song, Chunling Du, Zhihong Chen

**Affiliations:** 1 Respiratory Division of Zhongshan Hospital, Shanghai Institute of Respiratory Disease, Fudan University, No. 180 Fenglin Road, Shanghai, China; 2 Research Center of Zhongshan Hospital, Fudan University, Shanghai, China; 3 Department of Respiratory Medicine, Second Affiliated Hospital of Chongqing Medical University, Chongqing, China; 4 Department of Laboratory Medicine, Huashan Hospital, Shanghai Medical College, Fudan University, Shanghai, China; 5 Respiratory Division of Qingpu Hospital Affiliated to Zhongshan Hospital Fudan University, Shanghai, China

**Keywords:** Severe asthma, Oxidative stress, Phosphoinositide-3-kinase

## Abstract

**Background:**

Glucocorticoid (GC) insensitivity is an important feature of severe and fatal asthma. Oxidative stress can induce phosphoinositide-3-kinase (PI3K) activation, contributing to the development of GC insensitivity in chronic airway diseases. However, the underlying molecular mechanism of PI3K in the pathogenesis of severe asthma remains unknown.

**Methods:**

We isolated peripheral blood mononuclear cells (PBMCs) from 34 participants (12 patients with mild/moderate asthma, 10 patients with severe asthma, and 12 control subjects). H_2_O_2_ was used to stimulate the human macrophage line U937 to mimic the oxidative stress status in severe asthma. The ability of candidate compounds, namely, azithromycin, PI3K inhibitors (BEZ235 and LY294002) and a p38 MAPK inhibitor (BIRB796), to ameliorate GC insensitivity in severe asthma was evaluated.

**Results:**

PBMCs from patients with severe asthma exhibited dose-dependent and time-dependent GC insensitivity, which correlated with reduced activity of histone deacetylase 2 (HDAC2) (p < 0.05) and elevated expression of proinflammatory genes [nuclear factor-κB (NF-κB) and activator protein-1 (AP-1)] (p < 0.01) compared with these parameters in the control group. The PI3K inhibitors (BZE235 and LY294002) significantly restored the GC sensitivity of PBMCs from patients with severe asthma. In vitro, the PI3K inhibitors (BZE235 and LY294002) ameliorated GC insensitivity in H_2_O_2_/TNFα-induced IL-8 release from U937 cells by independently restoring the activity of HDAC2 or inhibiting the activation of transcription factors.

**Conclusions:**

This study demonstrates that PI3K inhibitors ameliorate GC insensitivity in severe asthma by restoring HDAC2 activity and inhibiting the phosphorylation of nuclear signaling transcription factors.

## Background

Approximately 5–10% of patients with asthma exhibit severe asthma and either do not respond well to glucocorticoid (GC) treatment or require high doses of inhaled or oral GCs to control asthma symptoms. These issues cause considerable difficulty in disease management and increase the financial burden, as few effective alternative treatments are available [1].

GCs exert their anti-inflammatory effects mainly via transrepression of proinflammatory genes [2]. GCs cross the cell membrane and bind to glucocorticoid receptors (GRs), and the GC/GR complex then translocates into the nucleus. The homodimer binds to glucocorticoid response elements (GREs) in the promoter region of corticosteroid-responsive anti-inflammatory genes, increasing anti-inflammatory gene transcription. Alternatively, the GC/GR complex may recruit corepressors, such as histone deacetylase 2 (HDAC2), and then directly interact with proinflammatory DNA-binding transcription factors (TFs), such as nuclear factor-κB (NF-κB) and activator protein-1 (AP-1). After the protein complex binds to the promoter region of a proinflammatory gene, it removes acetyl moieties from the amino terminal (NH) tails of the core histones and recondenses DNA around the core histone proteins, ultimately suppressing proinflammatory gene expression [3–5]. In addition, HDAC2 can deacetylate other transcriptional regulators, such as GR itself, and subsequently transrepress GC-mediated NF-κB gene expression [4].

Numerous studies have demonstrated possible mechanisms of GC insensitivity, such as genetic susceptibility, defective GR binding and nuclear translocation, increased glucocorticoid receptor β (GRβ) expression, transcription factor activation, abnormal histone acetylation and immune-related mechanisms [4, 6–8]. A recent study confirmed that interleukin-13 (IL-13)^+^ type 2 innate lymphoid cells (ILC2s) in peripheral blood are associated more strongly than Th2 cells with asthma severity and GC resistance in humans [9]. Interleukin-2 (IL-2) and interleukin-4 (IL-4) are overexpressed in the airways of patients with corticosteroid-resistant asthma [10]. The mechanism whereby these cytokines reduce GR function appears to be mediated through the phosphorylation of GR through p38MAPK, and this effect was blocked by treatment with a p38MAPK inhibitor [11]. Selective p38MAPK inhibitors increase the responsiveness to corticosteroids in alveolar macrophages and peripheral blood mononuclear cells (PBMCs) from patients with severe asthma [12]. Selective p38MAPK inhibitors are currently in clinical development for severe asthma, but the dose is limited by side effects after oral administration [13].

Molecular mechanisms underlying the reduction in HDAC2 expression in severe asthma have been elucidated [14]. Oxidative stress results in the formation of peroxynitrite, which nitrates tyrosine residues on HDAC2, resulting in its inactivation, ubiquitination, and degradation [15, 16]. In addition, oxidative stress activates phosphoinositide 3-kinase δ (PI3K δ), which leads to the phosphorylation and inactivation of HDAC2 [17]. This observation suggests that oxidative stress is a major mechanism leading to GC insensitivity in patients with severe asthma [18]. In vivo studies demonstrated a role for oxidative stress in GC insensitivity in a mouse model of cigarette smoke-induced airway inflammation, but this was debilitated by a specific inhibitor of PI3Kδ [11]. In addition, both PI3Kδ and γ are proposed to be anti-inflammatory targets [19, 20], because PI3Kδ is implicated in B and T cell signaling, mast cell-mediated allergic responses and neutrophil activation, whereas PI3Kγ is linked to neutrophil activation and mast cell degranulation [21, 22]. However, the precise molecular mechanism of PI3K in the pathogenesis of severe asthma remains unclear. Macrolides, including nonantibiotic macrolides, also reverse corticosteroid resistance through the inhibition of PI3K pathways but might act more distally in the pathways [23].

Herein, we used PBMCs isolated from asthmatic patients and established a cell model named H_2_O_2_-TNFα-U937 to mimic oxidative stress-induced GC insensitivity in vitro. Several compounds were screened to select appropriate targets as potential add-on reagents for improving GC sensitivity. In addition, the underlying biochemical and molecular mechanisms were investigated.

## Methods

### Study participants

All subjects were recruited from the Pulmonary Division of Zhongshan Hospital, Fudan University, China, with approval by the local ethics committee of Zhongshan Hospital of Fudan University. Written informed consent was obtained before screening. The study enrolled 12 participants with mild/moderate asthma, 10 participants with severe asthma, and 12 healthy control participants (Table 1). Participants were eligible if they had asthma with a positive bronchial provocation test or bronchial dilation test. Severe asthma was defined as the requirement for high medication doses to maintain good symptom control or the presence of persistent symptoms, asthma exacerbations, or airflow obstruction despite high medication doses [24]. Exclusion criteria included a history of smoking, chronic obstructive pulmonary disease (COPD), bronchiectasis, pulmonary fibrosis or hereditary hormone deficiency disorders. No subject used rifampicin, erythromycin, anticonvulsants or other drugs that may affect the efficiency of hormones in the last month before screening. The healthy control participants had no medical history of asthma, allergic rhinitis or other allergic diseases. Pulmonary function tests were performed as previously described. Approximately 15 ml of peripheral venous blood was extracted from each subject between 6 a.m. and 9 a.m. using 10% EDTA as an anticoagulant.

**Table 1:** Demographic and clinical characteristics of participants with severe or mild/moderate asthma and control subjects

	Mild/moderate asthma (n = 12)	Severe asthma (n = 10)	Healthy control (n = 12)
Sex (male vs. female)	5 vs. 7	4 vs. 6	6 vs. 6
Age (years)	45.0 ± 13.9	51.8 ± 12.0	48.2 ± 12.5
History (years)	15.3 ± 10.9	11.9 ± 12.4	ND
Allergic rhinitis (n, %)	8 (66.7%)	6 (60.0%)	0 (0%)
FEV1% pre (mean ± SD)	75.2 ± 12.7	63.2 ± 11.3*	101 ± 15.5
FEV1/FVC (mean ± SD)	83.7 ± 16.8	73.5 ± 18.5*	97.9 ± 17.2
Bronchodilator responsiveness positive (n, %)	12 (%)	10 (%)	ND
FeNO (ppb) (mean ± SD)	38.9 ± 24.9	75.6 ± 21.5*	19.2 ± 5.7
Eosinophils (× 10^9^/l) (mean ± SD)	0.27 ± 0.25	0.35 ± 0.18	ND
IgE (IU/ml) (mean ± SD)	263.9 ± 400.6	303.7 ± 261.2	ND
ACQ (mean ± SD)	5.7 ± 1.5	11.9 ± 2.9*	ND
ACT (mean ± SD)	22.9 ± 6.8	16.8 ± 5.1*	ND
≥ 1 Acute exacerbation during the previous year (n, %)	2 (16.7%)	6 (60.0%)	ND

*FEV1% pre* forced expiratory volume in 1 s, *FVC* forced volume capacity, *FeNO* fraction of exhaled nitric oxide, *ACQ* Asthma Control Questionnaire, *ACT* asthma control test, *ND* not detected

*p < 0.05 (compared to mild/moderate asthma)

### Reagents and antibodies

The following reagents were used: dexamethasone (Sigma, Dorset, UK), H_2_O_2_ (Sigma, Dorset, UK), azithromycin, LY294002, BEZ235 (Selleck, Houston, USA), and tumor necrosis factor α (TNFα) (Sigma, Dorset, UK). The following antibodies were used: goat anti-rabbit (Abcam, Cambridge, UK), anti-phosphorylated NF-κB (Cell Signaling Technology, Danvers, USA), anti-phosphorylated c-Jun (Cell Signaling Technology, Danvers, USA), and anti-phosphorylated c-Fos (Cell Signaling Technology, Danvers, USA).

### PBMC isolation and culture

Approximately 15 ml of peripheral venous blood was extracted from each subject between 6 a.m. and 9 a.m. using 10% EDTA as an anticoagulant. Isolation of human PBMCs was performed as previously described [7]. Fifteen milliliters of venous blood was diluted with 20 ml of sterile Hank’s balanced salt solution (HBSS) in a 50 ml tube. Thirty-five milliliters of diluted blood was slowly layered on top of 12.5 ml of Ficoll-Paque and centrifuged for 30 min at 400×*g* and room temperature. The mononuclear cells at the interface of the upper layer and the Ficoll-Paque were collected into a new tube, diluted with HBSS, and centrifuged for 10–15 min at 400×*g* and 4 °C. Cells were resuspended in HBSS and were then washed twice with phosphate-buffered saline (PBS). Cells were pelleted by centrifugation at 400×*g* for 10–15 min at 4 °C and resuspended in 5 ml of medium. Kimura-stained cells were counted on a hemocytometer and resuspended at 5 × 10^6^ cells/ml. Viability was assessed using trypan blue.

PBMCs obtained from asthma patients and healthy controls were cultured in RPMI 1640 medium (HyClone, Logan, USA) supplemented with 10% fetal calf serum (Gibco, Grand Island, USA) and 1% antibiotics (penicillin: 100 U/ml, streptomycin: 100 μg/ml) at 37 °C in a humidified atmosphere containing 5% CO_2_.

### TNFα-induced IL-8 production in cell culture

In the dose–response assay, cultured PBMCs were pretreated with different concentrations of dexamethasone (0.01 μM, 0.1 μM, 1 μM, and 10 μM) or PBS (control group) 4 h before stimulation with 0.01 ng/ml TNFα. Supernatants were collected after 24 h of incubation, and IL-8 release was measured by enzyme-linked immunosorbent assay (ELISA). In the time course assay, PBMCs were pretreated with 0.01 ng/ml TNFα and were subsequently treated with 0.1 μM dexamethasone 1 h, 2 h, 4 h, and 6 h later. Supernatants were collected after overnight incubation, and IL-8 release was measured by ELISA.

### Compound selection

To determine whether add-on compounds can ameliorate GC insensitivity in severe asthma, azithromycin, PI3K inhibitors (BEZ235 and LY294002) and a p38 MAPK inhibitor (BIRB796) were used. PBMCs were isolated from patients with severe asthma and pretreated with different concentrations of dexamethasone (0 µM, 0.01 μM, 0.1 μM, and 1 μM), and azithromycin, a PI3K inhibitor (BEZ235) and a p38 MAPK inhibitor (BIRB796) were then added separately to the combination treatment groups. Forty-five minutes later, cells were stimulated with 0.01 ng/ml TNFα. After overnight incubation, IL-8 release was measured by ELISA.

### Measurement of HDAC2 activity in PBMCs

First, nuclear extracts were prepared using a nuclear extraction kit (EpiGentek, USA). Nuclear extracts can be used immediately to detect the activity of HDAC2 or frozen at − 80 °C for future use. An HDAC2 activity assay kit (EpiGentek, USA) was applied to analyze the HDAC2 activity for each sample according to the protocol.

### H_2_O_2_-TNFα-U937 cell model

U937 cells (ECACC code 85011440) were cultured at 37 °C in a humidified atmosphere containing 5% CO_2_ in RPMI 1640 medium containing 10% fetal calf serum, 100 U/ml penicillin, and 100 μg/ml streptomycin at a density of 1 × 10^6^ cells/ml. Based on the preliminary experiment (Additional file 1), cells were stimulated with 400 μM H_2_O_2_ in RPMI medium for 4 h, and 0.1 μM dexamethasone was then added 45 min before incubation with TNFα (0.01 ng/ml). After overnight incubation, IL-8 release was measured using ELISA kits (R&D Systems) according to the manufacturer’s instructions.

### Real-time PCR (RT-PCR)

The PCR primers were as follows IL-8: (F) 5′-TTGCCAAGGAGTGCTAAAGAA-3′, (R) 5′-GCCCTCTTCAAAAACTTCTCC-3′; GAPDH: (F) 5′-CCACCCATGGCAAA TTCCATGGCA-3′, (R) 5′-TCTACACGGCAGGTCAGGTCCACC-3′. Primers were purchased from R&D Systems and used according to the manufacturer’s instructions. Extraction of RNA from PBMCs and U937 cells was performed by using a RNeasy Mini Kit according to the manufacturer’s instructions (Qiagen, Crawley, UK). Sample RNA was quantified by spectrophotometry, and 1 μg was reverse transcribed to cDNA as previously described [11]. PCRs were performed on 2 μl of cDNA in a Hybaid Omnigene thermal cycler (Hybaid, Ashford, Middlesex, UK) in a final reaction volume of 20 μl in the presence of 0.4 U of Taq DNA polymerase. Forty cycles with a denaturation step at 98 °C for 5 min followed by an annealing step at the specific primer annealing temperature for 15 min were used.

### Western blot analysis

Treated cells were lysed in radioimmunoprecipitation assay (RIPA) buffer (Millipore, Billerica, MA, USA) and boiled at 100 °C for 5 min with 4 × protein loading dye (8% SDS, 0.04% Coomassie Blue R-250, 40% glycerol, 200 mM Tris–HCl (pH 6.8), and 10% 2-mercaptoethanol). Samples were then subjected to sodium dodecyl sulfate–polyacrylamide gel electrophoresis (SDS-PAGE). Proteins were transferred to a polyvinylidene difluoride (PVDF) membrane (Sartorius Stedim, Aubagne Cedex, France) and incubated with primary antibodies against specific proteins (phosphorylated (p-)-NF-κB, p-c-FOS, p-c-JUN or GAPDH) at 4 °C overnight (1:2000 dilutions). Membranes were then incubated with a goat anti-rabbit secondary antibody (1:10,000 dilution) and analyzed using a Chemiluminescent ECL Detection System (Merck Millipore, Billerica, MA, USA). The protein levels were normalized to the GAPDH level. The signal intensity was quantified using Vision Works LS 6.3.3 (UVP, Cambridge, UK).

### ELISA

The concentration of IL-8 was determined using commercially quantitative ELISA kits (R&D, Minnesota, USA) according to the manufacturer’s instructions. The plate was coated with 100 μl of IL-8 capture antibodies overnight at 4 °C, blocked at room temperature for 1 h and incubated with 100 μl of the sample at room temperature for 2 h. The detection antibody was added and incubated at room temperature for 2 h. Finally, 50 μl of termination solution was added to each well, and the absorbance was read in a microplate reader at 450 nm.

### Statistical analysis

For all experiments, statistical analysis was first performed for all groups using one-way ANOVA to determine statistically significant variances between groups for each endpoint assessed. Statistical significance between groups was then assessed using a t test (Mann–Whitney test). All statistical analyses were performed using SPSS 21.0 software. Data are expressed as the means ± SEMs. Differences were considered significant at p < 0.05.

## Results

### PBMCs from patients with severe asthma demonstrated dose-dependent and time-dependent dexamethasone insensitivity

**Fig. 1:**
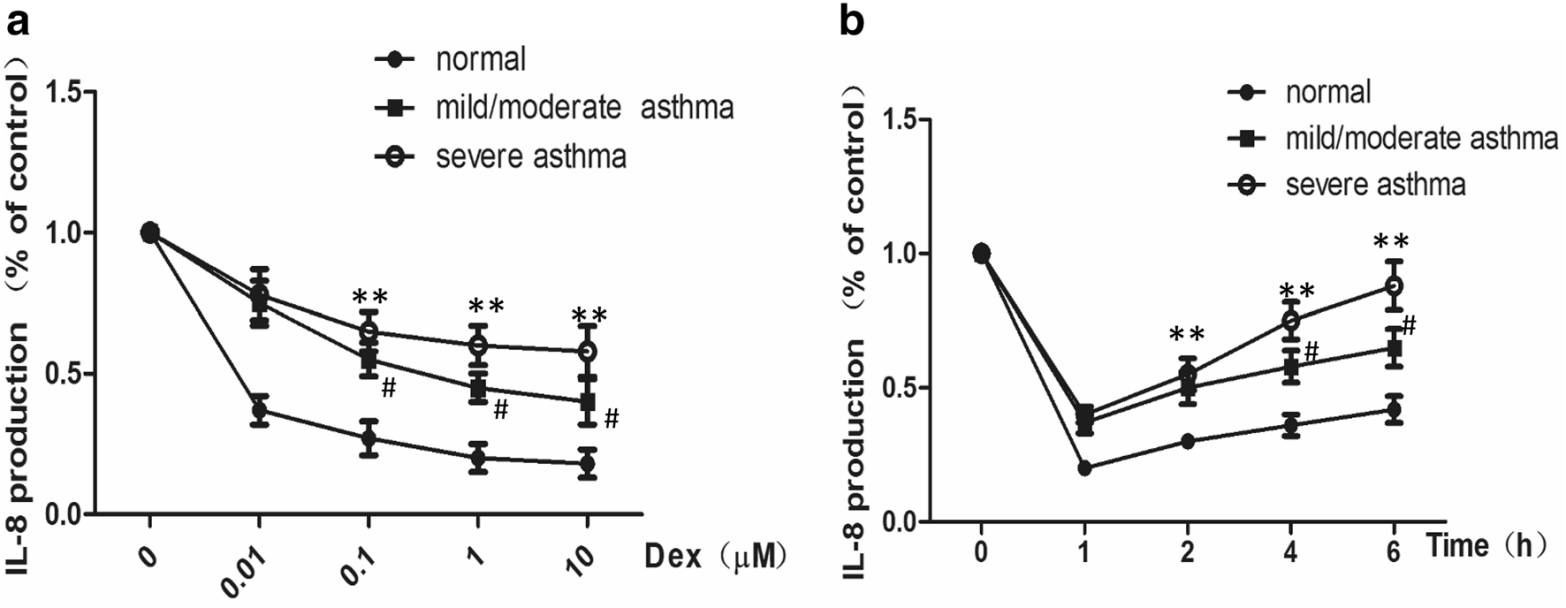
PBMCs with severe asthma showed dexamethasone insensitivity in the TNFα-induced IL-8 release test. **a** Dexamethasone inhibits IL-8 releasing of PBMCs in dose-dependent manner, which is impaired in asthma population, especially in severe asthma subgroup. **b** Dexamethasone added 1–6 h after TNFα stimulation. It showed dexamethasone inhibited- IL-8-releasing gradually diminished in a time-dependent manner. Dexamethasone inhibition rate decreased in asthma population, especially in severe asthma subgroup. IL-8 releasing level is relative to the level of group without dexamethasone treatment. **p < 0.01 significant difference when compared to severe asthma group; ^#^p < 0.05 significant difference when compared to severe asthma group

To compare the effect of dexamethasone on TNFα-induced IL-8 release, PBMCs from the healthy control, mild/moderate asthma and severe asthma groups were pretreated with different concentrations of dexamethasone and were then stimulated with 0.01 ng/ml TNFα. In the time course assay, dexamethasone was added at time points from 1 to 6 h after TNFα stimulation, and these results showed dexamethasone inhibited IL-8 release from PBMCs in both a dose-dependent and time-dependent manner in the healthy control group (p < 0.01, Fig. 1). The inhibitory effect of dexamethasone increased with gradually increasing concentrations. However, the effect was significantly decreased in asthmatic patients, especially in the severe asthma subgroup, indicating the dexamethasone insensitivity of PBMCs from patients with severe asthma.

### Reduced activity of HDAC2 and elevated expression of proinflammatory genes in severe asthma

**Fig. 2:**
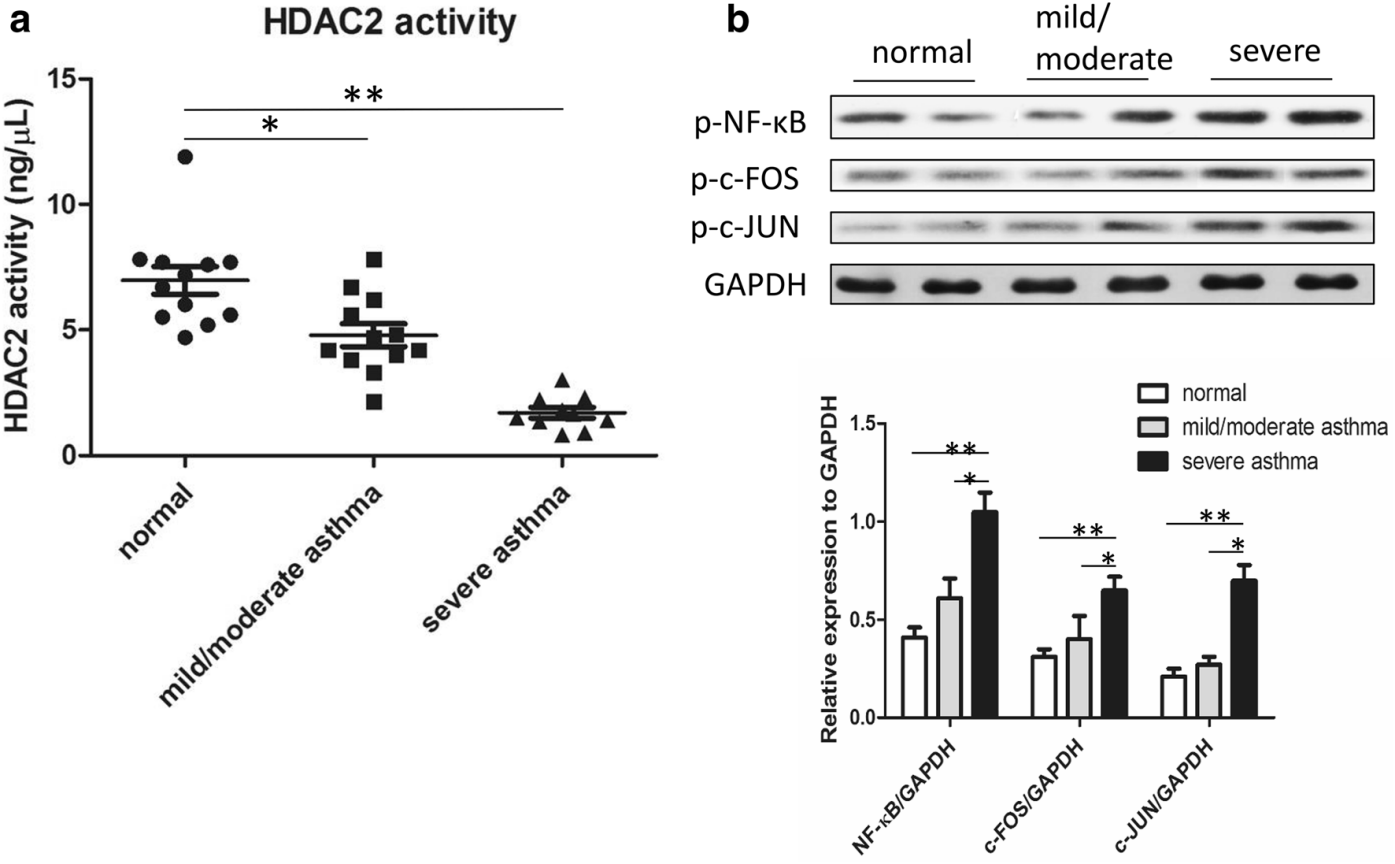
Molecular mechanisms underlying the dexamethasone insensitivity of PBMCs in severe asthma. **a** HDAC2 activity in PBMCs from normal controls and patients with mild/moderate or severe asthma [*p < 0.05; **p < 0.01, n = 12, 12, 10 (normal, mild/moderate asthma, severe asthma, respectively)]. **b** The gene expression of nuclear signaling transcription factors (p-NF-κB, p-c-FOS, p-c-JUN) in PBMCs from normal controls and patients with mild/moderate or severe asthma was analyzed by western blotting. The lower right panel shows the quantitative densitometric analysis. Representative data from at least 3 independent experiments are shown [*p < 0.05; **p < 0.01; n = 12, 12, 10 (normal, mild/moderate asthma, severe asthma, respectively)]

The anti-inflammatory effects of GCs arising via the transrepression of proinflammatory genes are mediated predominantly through glucocorticoid receptor-α (GR-α). GR-α transrepression is dependent on the recruitment of HDAC2 and subsequent termination of gene expression through the deacetylation of core histone proteins and recondensation of DNA. Reduced HDAC2 activity is proposed to play a role in chronic inflammation and GC insensitivity. In this study, we extracted the nuclear protein fraction from PBMCs obtained from normal subjects and asthmatic subjects (mild/moderate and severe) and measured HDAC2 protein activity with an HDAC2 assay kit. HDAC2 protein activity was significantly lower in the asthma group than in the normal group (p < 0.05) and markedly lower in the severe asthma group than in the mild/moderate asthma group (p < 0.01) (Fig. 2a).

Proinflammatory genes are generally activated by transcription factors, particularly NF-κB and AP1, in the airways of patients with asthma. Structurally, AP-1 is a heterodimer composed proteins belonging to the c-Fos, c-Jun, and other families. In this study, the levels of transcription factors, including phosphorylated NF-κB, c-FOS, and c-JUN, was measured by western blot. The results showed gradually elevated expression of p-NF-κB, p-c-FOS, and p-c-JUN from normal subjects to patients with mild/moderate asthma to patients with severe asthma (p < 0.01 severe asthma vs. normal; p < 0.05 severe asthma vs. mild/moderate asthma) (Fig. 2b).

### Screening compound candidates as add-on therapeutic reagents for improving dexamethasone sensitivity in severe asthma

**Fig. 3:**
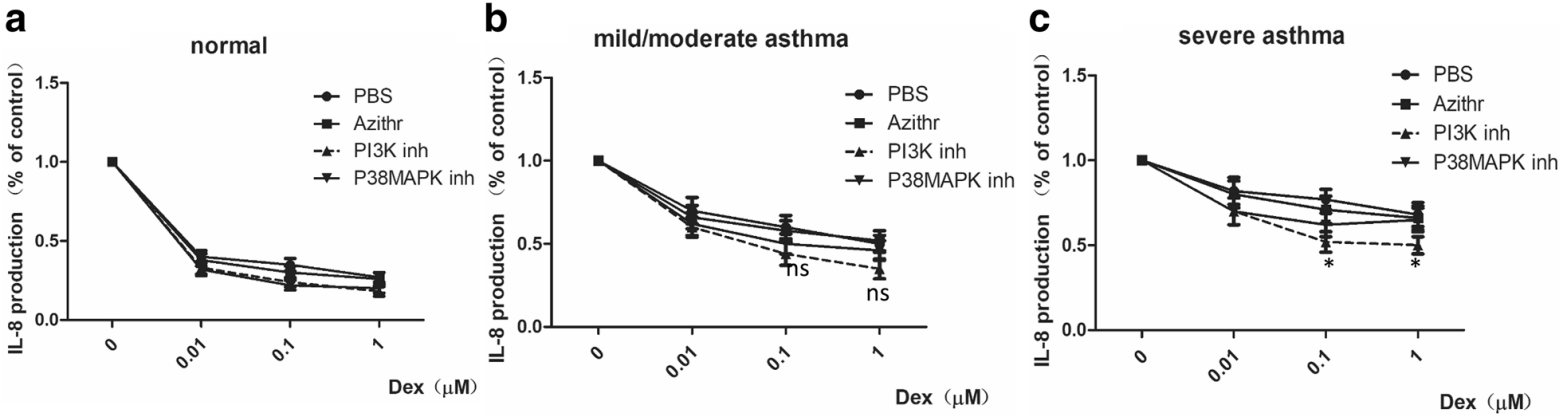
Add-on compounds to reverse the dexamethasone insensitivity of PBMCs in severe asthma. **a** The inhibition of IL8 release in PBMCs of normal subjects when compound was added to dexamethasone (0.01 μM, 0.1 μM, and 1 μM) treated medium. **b** The inhibition of IL8 release in PBMCs of mild/moderate asthma when compound was added to dexamethasone (0.01 μM, 0.1 μM, and 1 μM) treated medium. **c** The inhibition of IL8 release in PBMCs of severe asthma when compound was added to dexamethasone (0.01 μM, 0.1 μM, and 1 μM) treated medium (IL-8 releasing level is relative to the level of group without dexamethasone treatment. *p < 0.05, PI3K inhibitor added group compared with PBS added group. ns: no significant difference, PI3K inhibitor added group compared with PBS added group)

Azithromycin, a PI3K inhibitor and a P38MAPK inhibitor were selected as candidate compounds. PBMCs from normal subjects and asthmatic subjects (mild/moderate and severe) were pretreated with dexamethasone (0.01 μM, 0.1 μM, and 1 μM) plus the individual compounds [5 μM azithromycin, 1 μM BEZ235 (PI3K inhibitor), and 5 μM BIRB796 (P38MAPK inhibitor)] for 4 h. A group treated with PBS plus dexamethasone was established as a control group. Cells were stimulated with 0.01 ng/ml TNFα overnight. The supernatants were collected for an IL-8 ELISA. The results confirmed that 0.1 μM or 1 μM dexamethasone plus a low dose of BEZ235 (1 μM), a PI3K inhibitor, significantly restored the corticosteroid sensitivity of PBMCs from patients with severe asthma (p < 0.01) (Fig. 3c). BIRB796 (5 μM), a P38MAPK inhibitor, also reversed dexamethasone insensitivity in PBMCs from patients with severe asthma in 0.1 μM dexamethasone pretreated subgroup (p < 0.01) (Fig. 3c). Azithromycin had no obvious effect compared to PBS treatment in PBMCs from patients with severe asthma (p = 0.07) (Fig. 3c).

### PI3K inhibitor add-on treatment reversed dexamethasone insensitivity in the H_2_O_2_-TNFα-U937 cell model

**Fig. 4:**
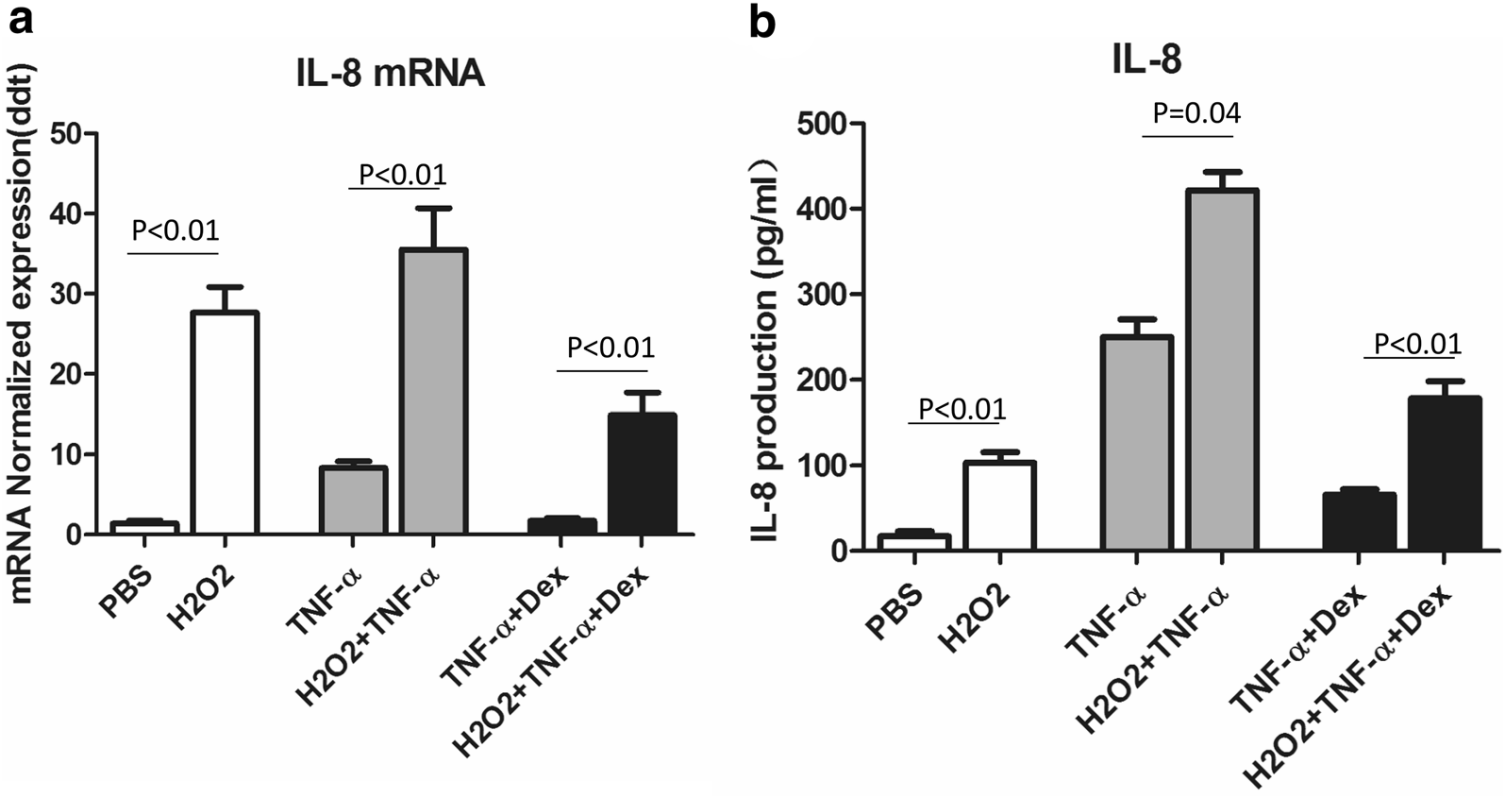
Establishment of the H_2_O_2_-TNFα-U937 cell model to mimic the dexamethasone insensitivity of PBMCs in severe asthma. **a** IL-8 mRNA expression in cells treated with H_2_O_2_, TNFα + H_2_O_2_, TNFα + DEX, or TNFα + H_2_O_2_ + DEX was measured by qPCR. **b** The IL-8 concentration in cells treated with H_2_O_2_, TNFα + H_2_O_2_, TNFα + DEX, or TNFα + H_2_O_2_ + DEX was measured by ELISA (p < 0.05, statistically significant. Each experiment was repeated at least three times)

Oxidative stress is present in several airway diseases, such as severe asthma and COPD, and contributes to the low responses to GCs through the downregulation of histone deacetylase (HDAC) activity. We used H_2_O_2_ to stimulate the human macrophage line U937 to mimic the oxidative stress status of PBMCs from patients with severe asthma. U937 cells incubated with H_2_O_2_ released more IL-8 than those treated with PBS or TNFα alone. Increased levels of IL-8 were released when cells were treated with TNFα plus H_2_O_2_. Dexamethasone obviously inhibited TNFα-induced IL-8 release, but the inhibition rate was relatively lower when TNFα plus H_2_O_2_ was added to the cells (Fig. 4). These results indicated that H_2_O_2_ treatment impaired the dexamethasone sensitivity of U937 cells (Fig. 4).

**Fig. 5:**
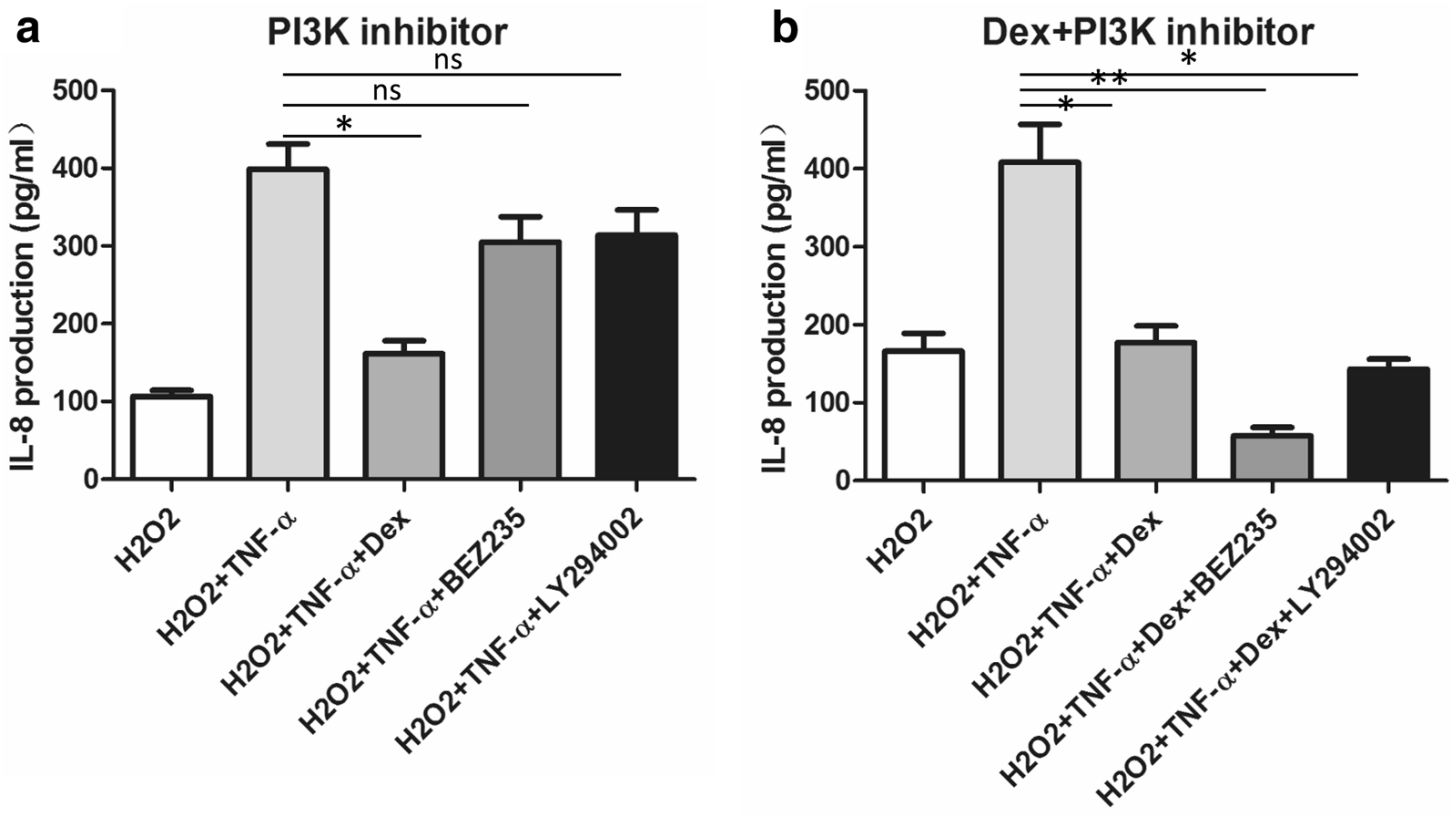
The effect of the PI3K inhibitor alone and in combination with dexamethasone on IL-8 release in the H_2_O_2_-TNFα-U937 cell model. **a** The IL-8 concentration was upon the addition of dexamethasone, BEZ235, and LY294002 was measured by ELISA. **b** The IL-8 concentration upon the addition of DEX + BEZ235 or DEX + LY294002 was measured by ELISA (*p < 0.05; **p < 0.01, ns: nonsignificant. Each experiment was repeated at least three times)

Next, we observed the effect of a PI3K inhibitor on IL-8 production in the H_2_O_2_-TNFα-U937 cell model. BEZ235 and LY294002 are two kinds of PI3K inhibitors. First, they were added to the cell model separately but neither obviously inhibited IL-8 production at either the mRNA or protein level (p > 0.05 vs. TNFα + H_2_O_2_, respectively). Dexamethasone alone had a partial inhibitory effect on IL-8 production (p < 0.05). Finally, BEZ235 and LY294002 were used as add-on drugs with dexamethasone. The combinations showed an even greater inhibitory effect than dexamethasone treatment alone (Fig. 5).

### Combination treatment with a PI3K inhibitor (BEZ235) and dexamethasone reversed HDAC2 activity in the H_2_O_2_-U937 cell model

**Fig. 6:**
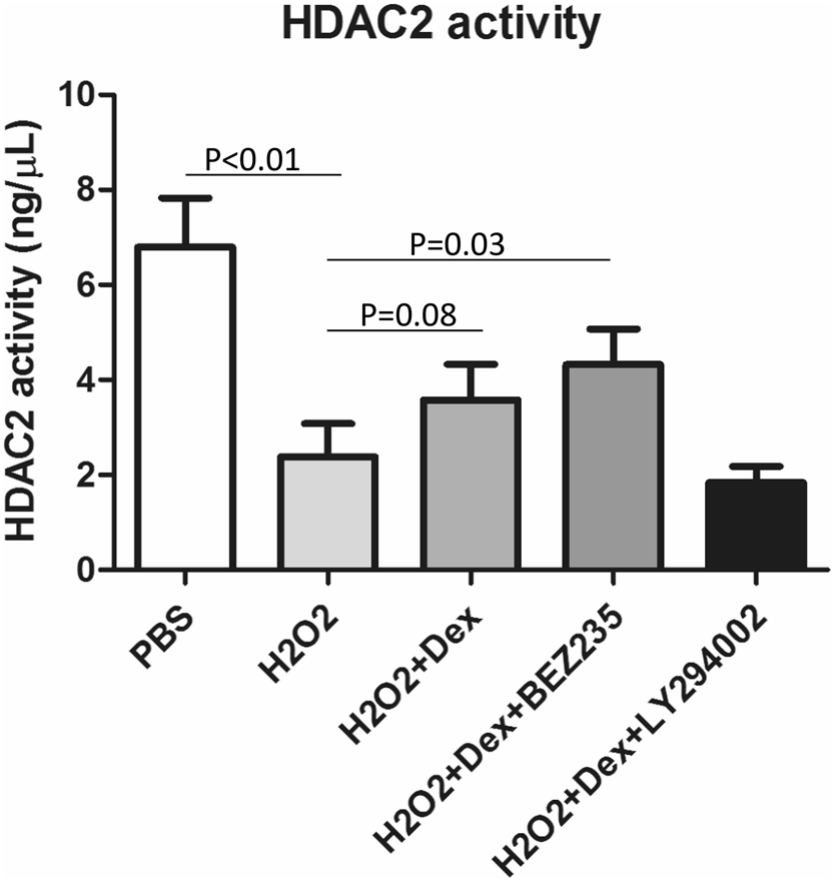
Dexamethasone alone and in combination with BEZ235 partially reversed HDAC2 activity in the H_2_O_2_-U937 cell model. The HDAC2 activity assay results showed that H_2_O_2_ markedly reduced HDAC2 activity. Dexamethasone alone or in combination with a PI3K inhibitor (BEZ235) partially reversed HDAC2 activity, especially in the combination treatment group (p < 0.05: statistically significant. Each experiment was repeated at least three times)

Oxidative stress can impair HDAC2 activity, which is implicated in the development of GC insensitivity. Consistent with this observation, nuclear HDAC2 activity was reduced in H_2_O_2_-treated U937 cells (p < 0.01) (Fig. 6). Dexamethasone treatment alone could not overcome the reduction in HDAC2 activity. However, treatment with the PI3K inhibitor BEZ235 plus dexamethasone significantly reversed HDAC2 activity in the H_2_O_2_-U937 cell model (p < 0.05) (Fig. 6).

### Effect of the PI3K inhibitors on the phosphorylation of nuclear signaling transcription factors in the H_2_O_2_-U937 cell model

**Fig. 7:**
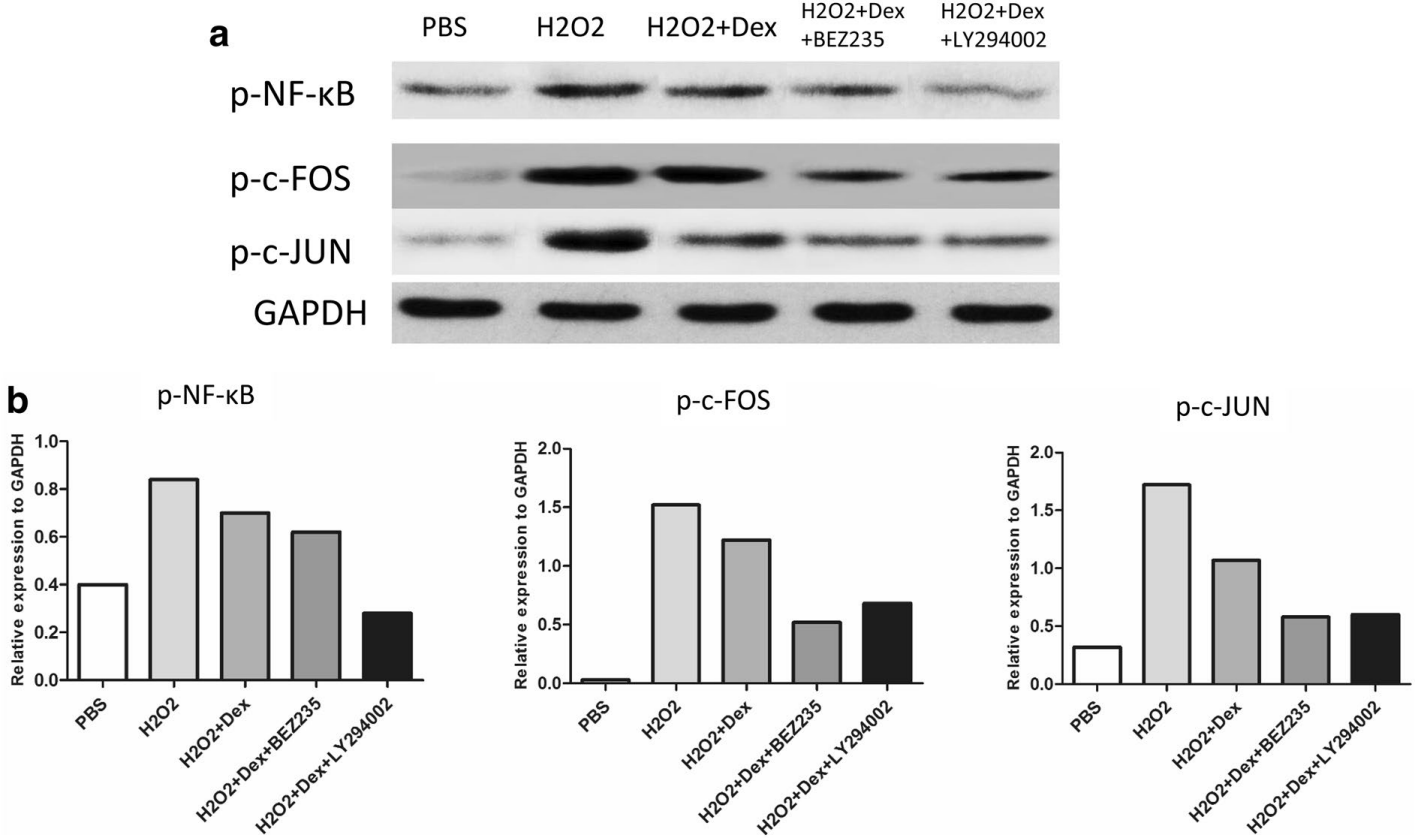
Effect of the PI3K inhibitor on the phosphorylation of nuclear signaling transcription factors in the H_2_O_2_-U937 cell model. **a** The expression of nuclear signaling transcription factors (p-NF-κB, p-c-FOS, and p-c-JUN) in the H_2_O_2_-U937 cell model was analyzed by western blotting. **b** Quantitative densitometric analysis of immunoreactive bands showed that p-NF-κB was most strongly inhibited by the combination of LY294002 and dexamethasone. p-c-FOS and p-c-JUN were most strongly inhibited by the combination of either BEZ235 or LY294002 with dexamethasone. Representative data from at least 3 independent experiments are shown (*p < 0.05; **p < 0.01, ns: nonsignificant. Each experiment was repeated at least three times)

To confirm the effect of the PI3K inhibitor on the phosphorylation of nuclear signaling transcription factors in the H_2_O_2_-U937 cell model, U937 cells were stimulated with 400 µM H_2_O_2_ in RPMI medium for 4 h and subsequently treated with 0.1 μM dexamethasone, 0.1 μM dexamethasone plus 1 μM BEZ235, and 0.1 μM dexamethasone plus 1 μM LY294002 for 24 h. Phosphorylation of nuclear signaling transcription factors (p-NF-κB, p-c-FOS, p-c-JUN) was measured by western blotting. H_2_O_2_ elevated the phosphorylation of nuclear signaling transcription factors (p-NF-κB, p-c-FOS, p-c-JUN) to a greater extent than observed in the control group (p < 0.01) (Fig. 7). Dexamethasone treatment had no significant effect on the p-NF-κB level. However, treatment with the PI3K inhibitor (LY294002) plus dexamethasone significantly decreased the p-NF-κB level (p < 0.01) (Fig. 7). The levels of p-c-FOS and p-c-JUN were significantly decreased by combination treatment with either BEZ235 or LY294002 and dexamethasone (p < 0.01) (Fig. 7).

## Discussion

Severe asthma accounts for a small number of asthma cases but has a high morbidity and mortality rate and is very costly to health care systems due to the reduced responsiveness to GCs and the requirement for long-term treatment. Airway inflammation is very heterogeneous in severe asthma. Higher eosinophil and neutrophil counts were found in induced sputum from patients with severe asthma than in that from patients with mild/moderate asthma. Emerging evidence has demonstrated that Th1/TC1 and Th17 can activate neutrophils and may play a role in this subtype of asthma. However, whether these inflammatory profiles are caused by environmental exposure to pollutants, smoking, infection, and other factors is unclear [25–28]. Interleukin-8 (IL-8), an inflammatory chemokine that activates neutrophils, is released by a wide range of cells, mainly human PBMCs and endothelial cells [29]. High expression of IL-8 in patients with severe asthma predicts poor asthma control. In our study, the ability of a GC (dexamethasone) to inhibit TNFα-induced IL-8 release was reduced in PBMCs obtained from patients with severe asthma compared with those obtained from patients with mild/moderate asthma or healthy subjects. The H_2_O_2_-TNFα-U937 cell model was established to mimic the oxidative stress status. The data showed that dexamethasone dramatically inhibited TNFα-induced IL-8 release, but the inhibitory effect was relatively lower in TNFα **+** H_2_O_2_-treated U937 cells.

The mechanisms underlying GC insensitivity in severe asthma are incompletely understood. Multiple mechanisms have been implicated in the pathogenesis of GC insensitivity in severe asthma. Some of these mechanisms include immune-mediated dysregulation of cytokines, defects in GR binding activity and translocation into the nucleus, increases in GRβ expression, excessive activation of AP-1 and NF-κB, and abnormal histone acetylation [30]. NF-κB is a transcription factor that plays an important role in proinflammatory responses. It exists in the cytoplasm bound to an inhibitory protein, IκB. Upon binding of cell signaling molecules such as interleukin-1β (IL-1β), IL-2, granulocyte–macrophage colony-stimulating factor (GM-CSF) and TNFα, a kinase cascade leads NF-κB to dissociate from IκB and translocate to the nucleus, where it drives the transcription of proinflammatory cytokines [31]. AP-1, a heterodimer of Fos and Jun, is a member of the leucine zipper transcription factor family that can enhance the transcription of other inflammatory genes. In addition, AP-1 has been demonstrated to physically interact with GR to prevent its binding to GREs [32]. In our study, the expression of transcription factors, including phosphorylated NF-κB, c-FOS and c-JUN, was higher in patients with severe asthma than in those with mild/moderate asthma or in healthy controls (Fig. 2b). Corticosteroids suppress transcription factor activities through the recruitment of HDAC2 [4] and switch off inflammatory genes, such as those encoding cytokines, chemokines, receptors and adhesion molecules, e.g., TNFα, IL-1β, IL-2, GM-CSF, IL-3, IL-6, IL-8, and IL-11 [33]. HDAC2 is markedly reduced in PBMCs and alveolar macrophages of patients with refractory asthma [7]. The results of our study are consistent with previously reported data and show that HDAC2 protein activity in the asthma group was significantly lower than that in the normal group and exhibited an even greater decrease between the severe asthma group and the mild/moderate asthma group (Fig. 2a).

In this study, several compounds—namely, azithromycin, a p38MAPK inhibitor and PI3K inhibitors, were selected as add-on therapies to reverse GC insensitivity in PBMCs from patients with severe asthma. Macrolides possess anti-inflammatory properties in addition to antimicrobial activity [34]. However, conflicting results have emerged from clinical trials. A study of 45 patients with severe asthma showed that compared with placebo, 8 weeks of clarithromycin treatment at a dosage of 500 mg twice daily significantly reduced airway neutrophilia and sputum IL-8 levels and significantly improved the Asthma Quality of Life Questionnaire (AQLQ) scores [35]. These differences were accentuated in the subgroup of patients with noneosinophilic asthma. Our study did not demonstrate an obvious effect of azithromycin add-on treatment on overcoming dexamethasone insensitivity in severe asthma in vitro (p = 0.07) (Fig. 3c), which was inconsistent with previous reports. The reason might be that macrolides specifically target specific subgroups of asthma.

GR function is reduced through phosphorylation by several kinases, most notably p38MAPK. Stress induces phosphorylation of serine 134 on GR in a p38MAPK-dependent manner [36], and this phosphorylation is relevant to GC insensitivity through the inhibition of nuclear translocation, protein stabilization, and DNA binding [30]. P38MAPK activity is inhibited by p38MAPK inhibitors, and this inhibition correlates with decreased phosphorylation of GR, resulting in increased nuclear translocation and GRE binding ability [12]. In vitro studies using PBMCs have examined the effects of p38 MAPK inhibition on steroid responsiveness. Dexamethasone plus inhibitor, compared with dexamethasone alone, can inhibit IL-8 release greatly. This finding suggests that these inhibitors may attenuate inflammatory responses in the presence of steroids [12]. In our study, we found that BIRB796, a P38MAPK inhibitor, reversed dexamethasone insensitivity in PBMCs from patients with severe asthma (p < 0.01) (Fig. 3c). However, a high dose of BIRB796 (10 μM)—tenfold higher than the dose used in other studies—had an inhibitory effect. Therefore, we did not select BIRB796 for further study (data not shown).

A recent report indicated that oxidative stress induces a proinflammatory state and GC insensitivity through the activation of PI3K [37]. PI3Kδ (a PI3K isoform) can be markedly upregulated in peripheral lung tissues of patients with COPD under oxidative stress [37, 38]. Activation of the PI3K pathway by oxidative stressors, such as cigarette smoke, has previously been reported in macrophage-like U937 cells [37]. In vivo, oxidative stress has been demonstrated to induce relative GC insensitivity in the airways of mice during cigarette smoke-induced inflammation, and COPD patients can be protected by specific inhibition of PI3Kδ [37]. As shown in our study, PI3K inhibitors (BEZ235 and LY294002) alone did not obviously inhibit IL-8 production in the H_2_O_2_-TNFα-U937 cell model. However, when combined with dexamethasone, these two subtypes of PI3K inhibitors effectively inhibited IL-8 release from H_2_O_2_-TNFα cells, which suggested that the PI3K inhibitors had a synergistic effect on overcoming GC insensitivity.

Oxidative stress is a major cause of COPD by reducing the expression and activity of HDAC2 and impairing corticosteroid action. Oxidative stress induces PI3K activation and decreases HDAC2 activity, which is thought to be closely related to the development of steroid insensitivity in COPD [37]. To date, however, the mechanism by which oxidative stress induces severe asthma through the regulation of HDAC2 activity has not been well illuminated. In asthmatic patients who smoke, tyrosine nitration of HDAC2 is inhibited, which appears to be an important mechanism of GC insensitivity in this subtype of asthma [39]. In fact, we showed here that H_2_O_2_ decreased the inhibitory effects of dexamethasone on TNFα-induced IL-8 release in U937 cells (Fig. 4). Similar to previous studies, ours showed that nuclear HDAC2 activity was reduced in H_2_O_2_-treated U937 cells (p < 0.01) (Fig. 6). Combination treatment with a PI3K inhibitor (BEZ235) and dexamethasone significantly reversed HDAC2 activity (Fig. 6). Interestingly, there was no difference between the two kinds of PI3K inhibitors. BEZ235, a selective PI3K inhibitor, reduced PI3K/AKT/mTOR signaling activity and greatly reversed HDAC2 activity at the same time, whereas LY294002, a nonselective PI3K inhibitor, reversed GC insensitivity by inhibiting the activation of transcription factors, such as NF-κB, c-FOS and c-JUN but did not target HDAC2.

Our study has several limitations, including the absence of downstream inflammatory signals other than IL-8. Other type 2 inflammatory cytokines, such as IL-4, IL-5, and IL-13, have not been detected in this system. Importantly, we selected the H_2_O_2_-TNFα-U937 system as a cell model to mimic oxidative stress-induced GC insensitivity, which was not a valid severe asthma model. Antigens that are frequently used in asthma models, such as ovalbumin and house dust mites, should be considered for use in further studies. Various immune cells in addition to monocytes (U937, a macrophage-like cell line) are involved in the pathogenesis of asthma. These cells also play role in severe asthma which deserve further investigation in the future.

## Conclusions

**Fig. 8:**
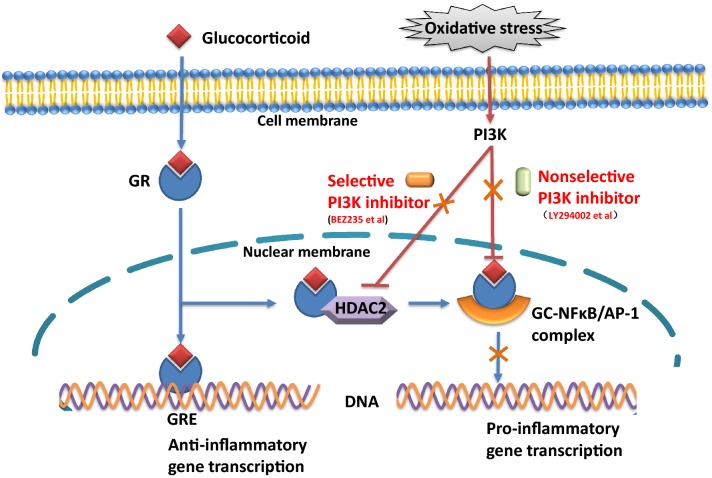
Possible mechanism by which PI3K inhibitors ameliorate GC insensitivity in severe asthma. GC bind with GC receptors (GR) in the cytoplasm,then the GC-bound GR complex diffuse across the nuclear membrane where it binds to the glucocorticoid response element (GRE). The GRE is responsible for transcribing anti-inflammatory proteins. Additionally, binding of GC to GR results in recruitment of histone deacetylase 2 (HDAC2), which is responsible for deacetylating GR, permitting its binding to nuclear factor-kappa B (NF-κB) and activating protein-1 (AP-1). Upon binding, these transcription factors are deactivated, thereby inhibiting the transcription of pro-inflammatory proteins. Additionally, HDAC2 deacetylates the histone permitting transcription of anti-inflammatory genes by GR. In severe asthma, oxidative stress can activate phosphoinositide 3-kinase (PI3K) pathway. PI3K phosphorylation reduce HDAC2 activity, affect the balance between pro-inflammatory and anti-inflammatory gene transcription and finally decrease corticosteroid sensitivity. Selective PI3K inhibitor (BEZ235 et al.) can restore HDAC-2 activity, and nonselective PI3K inhibitor (LY294002 et al.) can directly inhibit phosphorylation of nuclear signal transcription factors and finally improve glucocorticoid insensitivity

These data showed that treatment with a PI3K inhibitor plus dexamethasone can ameliorate GC sensitivity in severe asthma in vitro via a mechanism involving the restoration of HDAC2 activity and inhibition of phosphorylation of nuclear signaling transcription factors (Fig. 8). Clinically, PI3K inhibitors could be used as an add-on therapy for severe asthma.

## Supplementary information

**Additional file 1: Figure S1.** The viability of U937 cell under varied concentration of H_2_O_2_. The U937 cell viability measured by CCK-8 cell viability kit under varied concentration of H_2_O_2_. H_2_O_2_ with 200, 400 and 600 μM was used to treat U937 respectively and we found 400 μM H_2_O_2_ can stimulate cell to release more IL-8 than 200 μM H_2_O_2_. Next, the cell viability was tested using CCK-8 cell viability kit which showed the cell viability under the treatment of 400 μM H_2_O_2_ was 94%.

## Data Availability

The datasets supporting the conclusions of this article are included within the article.

## Abbreviations

GC: glucocorticoid
PI3K: phosphoinositol-3-kinase
PBMCs: peripheral blood mononuclear cells
HDAC2: histone deacetylase 2
NF-κB: nuclear factor-κB
AP-1: activator protein-1
GR: glucocorticoid receptor
ILC2: type 2 innate lymphoid cells
PBS: phosphate-buffered saline
TNFα: tumor necrosis factor α
RT-PCR: realtime PCR
ELISA: enzyme-linked immunosorbent assay
IL-8: interleukin-8
IL-1β: interleukin-1β
IL-2: interleukin-2
GM-CSF: granulocyte-macrophage colony-stimulating factor
AQLQ: Asthma Quality of Life Questionnaire
