# Supplementary material for: PI3K inhibitor treatment ameliorates the glucocorticoid insensitivity of PBMCs in severe asthma
Source: Clin Transl Med. 2020 Feb 28;9:22. doi: 10.1186/s40169-020-0262-5 (PMC7048898; doi:10.1186/s40169-020-0262-5)
Supplement: Supplementary file 1 — Additional file 1: Figure S1. The viability of U937 cell under varied concentration of H2O2. The U937 cell viability measured by CCK-8 cell viability kit under varied concentration of H2O2. H2O2 with 200, 400 and 600 μM was used to treat U937 respectively and we found 400 μM H2O2 can stimulate cell to release more IL-8 than 200 μM H2O2. Next, the cell viability was tested using CCK-8 cell viability kit which showed the cell viability under the treatment of 400 μM H2O2 was 94%. [file 40169_2020_262_MOESM1_ESM.pdf]

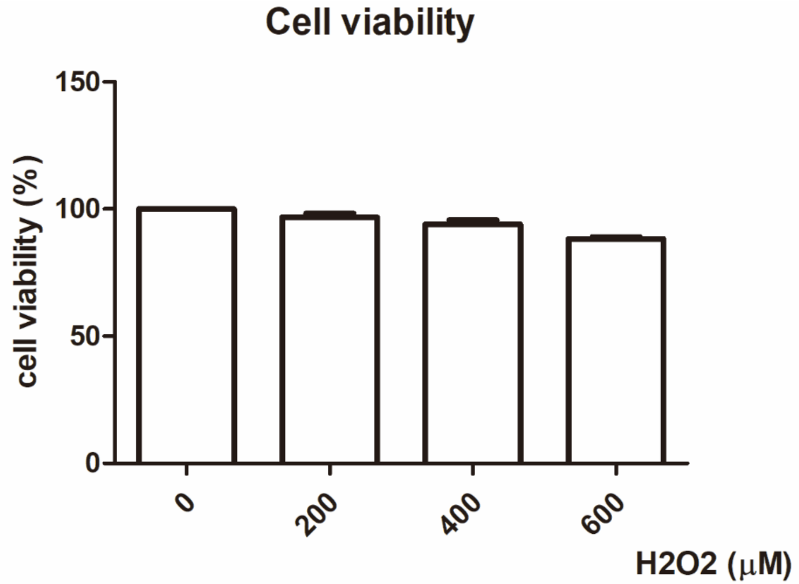

Additional file 1: Figure S1. The viability of U937 cell under varied concentration of H_2O_2. The U937 cell viability measured by CCK-8 cell viability kit under varied concentration of H_2O_2. H_2O_2 with 200, 400 and 600 μM was used to treat U937 respectively and we found 400μM H_2O_2 can stimulate cell to release more IL-8 than 200μM H_2O_2. Next, the cell viability was tested using CCK-8 cell viability kit which showed the cell viability under the treatment of 400μM H_2O_2 was 94%.
